# Localization of magnetocardiographic sources for myocardial infarction cases using deterministic and Bayesian approaches

**DOI:** 10.1038/s41598-022-25919-3

**Published:** 2022-12-21

**Authors:** Vikas R. Bhat, Basudha Pal, H. Anitha, Ananthakrishna Thalengala

**Affiliations:** 1 Department of Biomedical Engineering, Manipal Institute of Technology, Manipal Academy of Higher Education (MAHE), Manipal, India; 2 Department of Electronics and Communication Engineering, Manipal Institute of Technology, Manipal Academy of Higher Education (MAHE), Manipal, India

**Keywords:** Biomedical engineering, Cardiology, Mathematics and computing

## Abstract

In this paper, the inverse problems of cardiac sources using analytical and probabilistic methods are solved and discussed. The standard Tikhonov regularization technique is solved initially to estimate the under-determined heart surface potentials from Magnetocardiographic (MCG) signals. The results of the deterministic method subjected to noise in the measurements are discussed and compared with the probabilistic models. Hierarchical Bayesian modeling with fixed Gaussian prior is employed to quantify the uncertainties in source reconstructions. A novel application of Variational Bayesian inference approach has been presented to estimate the heart sources. The reconstruction results of Variational Bayesian model with non-stationary priors are compared with solutions of simplistic Bayesian approach; and the performances are evaluated using Root Mean Square Error (RMSE) and correlation co-efficient metrics. The Bayesian solutions in the study are also extended to localize the MCG sources for two types of Myocardial infarction cases.

## Introduction

The ionic exchange in the cardiac muscle cells creates a potential difference across the fibers known as transmembrane potentials. Due to this exchange, a tiny current flows throughout the heart cells in intra and extra cellular spaces. The spread of electrical activity on the myocardium follows a specific pathway to complete the cardiac cycle. The propagation of these electrical impulses help myocardial fibres to contract and relax. During the process of these contractions and expansions of muscles, the activity is manifested in terms of functional waves characterized as P, QRS and T waves^1^.

P shape is formed due to the contraction process of the atria and QRS is activated due to the ventricular depolarization; and the T wave is obtained from the relaxation of ventricles. The investigation of these electrophysiological changes in heart functioning is measured by Electrocardiogram (ECG) in terms of potential difference^2^. Any deviations in the ECG signals from the normal represent cardiac dysfunctions. Clinicians use ECG as a primary diagnostic tool to administer the functionality of heart diseases. Since this tool provides information at the surface level, sometimes clinicians may need further structural markings to confirm or localize the diseases with the help of non-invasive procedures.

In the biomedical society, researchers have contributed many works to minimize the invasive procedures in the diagnosis of heart diseases. Since the cardiac current flow is responsible for generating a tiny magnetic field across the human thorax, researchers^3,4^ developed a non-contact and non-invasive equipment called Magnetocardiogram (MCG) to record bio-magnetic fields.

MCG offers distinct advantages in analyzing the cardiac signals than ECG. Some advantages are as follows: (1) even though both ECG and MCG measurements are non-invasive, MCG is a non-contact procedure and requires less subject preparation than ECG. (2) The other advantage is that the problem of variations in skin-electrode contact impedance in ECG is circumvented in MCG due to the non-contact nature of the recordings^3^. (3) Also, the artefacts that may arise due to the fluctuating skin-electrode contacts in the ECG recordings are absent in the MCG. (4) MCG records the field due to primary currents and is least affected by the conductivity profiles of the intervening tissues present between the location of the heart and the thorax surface. This provides an ultimate scope to localize the cardiac sources more precisely within a few millimeters which is often not possible with the surface potentials measured in the ECG^5^.

However, these measurements can observe and record only at the surface levels and it would have been better for physicians to visualize the problems and diseases specifically at the heart level. Many researchers have contributed their works in non-invasively imaging the cardiac anomalies from the surface level recordings.

## Related work

Inverse problems have been solved in various applications, one of them is demonstrated by Qu et al.^6^ where authors analysed the steady state responses by solving the inverse problems based on Sparse-Bayesian learning.

Pullan et al.^7^, Hamalainen and Ilmoniemi^8^, Sarvas et al.^9^, contributed their works in modeling the heart activities non-invasively using electromagnetic inverse problems in the deterministic approaches.

Fang et al.^10^ proposed a novel framework to solve the ill posed inverse problems of spatiotemporal ECG using sparse decomposition and total variation method.

In order to solve the inverse problem, one has to model the structural heart and align it with the torso and detectors; and formulate a spatial relation between them using Maxwell’s equations, which is known as forward problem^11^. Tilg^12^, Huiskamp et al.^13^ mathematically assumed action potentials as distributed sources in the forward model. The authors^14^ used invasively pre-recorded epicardial potentials as cardiac sources and generated the spatial sensitivity in the forward study.

The inverse problem reconstructs the cardiac activation from the MCG signals along with the constructed transfer matrix. These bio-inverse problems are ill-posed since the sources are more in number than the sensors (in practice) and even small errors in the recordings may lead to large variations in the source estimations^9^. The common technique used to solve the inverse problem in deterministic approach is Tikhonov regularization (optimization methods)^13,14^. Mohammad-Djafari^15^ discussed and compared deterministic approach with the probabilistic model in the regularization theory and concluded that the latter method solved better in dealing with uncertainties and inaccuracies.

Zhou et al.^16^ demonstrated Sparse Bayesian Learning (SBL) (with Gaussian hierarchical priors) method to improve the localization accuracy of the inverse problem for the left-ventricular endocardium. The researchers reconstructed the endocardial potentials from the body surface ECGs and evaluated the results on the patient-specific and normal heart geometry models. The deterministic methods utilize some form of regularization, out of which the most widely used is Tikhonov^17^, which ensures the stable inverse solutions.

Serinagaoglu et al.^18^ evaluated the Bayesian approach to estimate the epicardial potentials from the body surface potentials. In this article, the authors solved the inverse ECG using surface measurements, a generic forward model with prior information of epicardial distributions (estimated from previous recordings) and the epicardial signals recorded invasively. The authors also discussed the advantages of Bayesian inference over deterministic methods. Bayesian framework includes error covariance that depends on the probability distribution of the prior sources and noise that effectively helps to yield the inverse solution.The other advantage of the Bayesian inference lies in the automatic estimation of the unknown posteriors by updating the covariance from the minimal knowledge about the hidden models. Whereas in Tikhonov method, one has to manually choose an optimal regularizer with the help of L-curve criterion to determine a good solution fit.

France^19^ studied Bayesian approach to quantify difference in *L*2 norm solutions that arise from conductivity and mesh discretization in the inverse problem of ECG. Even though the *L*2 norm solution provides satisfactory deterministic results, Bayesian not only estimate an equivalent maximum a-posteriori (MAP), but also capable of providing a distribution to study the sources of uncertainty. The Gaussian priors has been used to model the prior source activities.

The inverse problems addressed by Arinbjarnarson^20^ discuss for various types of Bayesian approaches in the context of Electroencephalography (EEG) domain. The Gaussian type models have been assumed to design the hyper-prior distributions in the inverse study.

The literature on Bayesian approach has been found to be confined to solving the bioelectric inverse problems; however, the framework could be extended to address the inverse problems in biomagnetism. This is the basic motivation to apply Bayesian modeling techniques for MCG.

The above mentioned Bayesian methods assume stationary Gaussian distribution and therefore estimate the unknown parameters from the observed measurements. The method fails to model non-stationary distributions.

Bishop^21^ explained about the competitive framework called Variational Bayes that imposes non-stationary distributions on the priors and hyper-prior models (distributions) to infer the unknowns with the help of Kullback–Leibler (KL) divergence. Tzikas et al.^22^ introduced Variational Bayesian Linear Regression (VBLR) approach where non-stationary Gaussian priors for hyperparameters were explored. The main idea of this method is to fit blue estimate the prior distribution with the help of varying family of distributions rather than a single prior in the simplistic Bayesian models. It has been reported that the solution quality of the VBLR approach is better than the stationary Bayesian models^23^.

Similar supporting work experimented by Rahimi et al.^24^ where a multiple-model Bayesian approach was proposed and utilized to solve the inverse problem of ECG data. The ill-posed nature was regularized by a fixed evaluation criteria which constrains the source distribution to follow a fixed prior structure rather than time varying sources. The issue has been addressed by multiple-prior models that uses time varying prior sources.The study was applied on the synthetic and real data experiments.

The experimental results demonstrated that the combination of different priors can be employed for assessing the complex source structures. Also, the proposed multiple model investigates the impact of different prior distributions which helps in reducing the poor fit of assumed model.

One of the objectives of this paper is to reconstruct epicardial sources using Bayesian frameworks to address the uncertainties that occur in the deterministic approach. The other objective is to apply Hierarchical models using the simplistic Bayesian and the novel (in this domain) Variational Bayesian methods in solving the inverse problem of epicardial activities. Further, the proposed inverse models have been analyzed based on noise-free and noisy MCG signals of normal and Myocardial Infarction cases.

## Methodology

In this section, the forward model of MCG has been described which discusses the source models, the explanation of the Lead Field/Transfer Matrix followed by the mathematical depiction of the forward model. In the next subsection, the inverse problem is described and solved using the standard Tikhonov method, the Hierarchical Bayesian method and the Variational Bayesian Linear Regression method. Two different heart models have been used, one for the forward problem and the other to solve the inverse problem in order to avoid the ‘inverse crime’.

### Forward problem

#### Source model

The source model is designed based on the prior knowledge of the electrophysiological nature of the heart. The source parameters assumed in the current study consisted of a ventricular surface model extracted from ECGsim^25^, covered by $Q = 257$ nodal locations with each discretized node assigned to epicardial potentials. The thorax model of size (25, 45, 45) cm consisting of 300 nodes with 596 tetrahedral meshes is considered in the study. The heart mesh was placed inside the thorax model at a location (0.3, 0.3,0) cm that lay in the realistic anatomical position between the lungs and behind the sternum^13^ (geometrical models were assembled in SCIRun software^26,27^). The volume of the thorax were filled with assumed conductivity $\sigma$ values of 0.6, 0.04 and 0.2 S/m for the ventricles, the lungs and the torso, respectively^28^.

**Figure 1:**
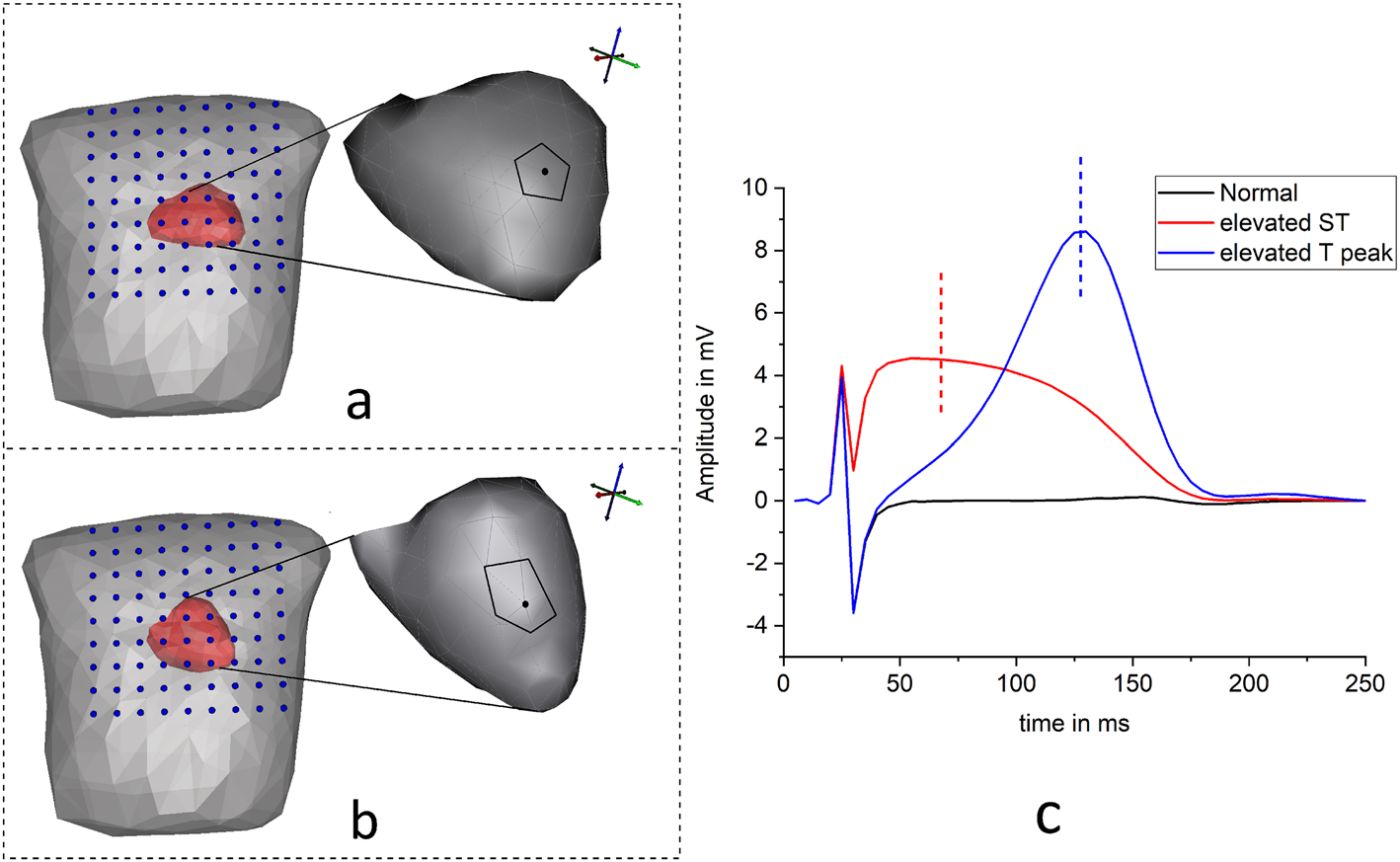
Source modeling and simulation: (**a**,**b**) Heart and Torso models aligned with MCG detectors (inset: ruptured cells patch considered in the study), (**c**) simulated epicardial potentials at the selected node (vertical lines indicate the desired time instants in ms).

The discretized model of the heart is defined as the vector of ${\textbf {q}} =({\textbf {q}}_1,\, {\textbf {q}}_2,\, \ldots {\textbf {q}}_Q)$ sources. The ventricular surface nodes at ${\textbf {q}}$ points oriented in $\hat{{\textbf {a}}}_{q}$ directions have been assigned with epicardial potentials. In this study, two types of Myocardial infarction (MI) cases are simulated using ECGsim software^25^. The activations are created at a node with 1.5 cm potential spread (shown as patch in the inset of Fig. 1a) near the anterior left ventricular wall by varying the transmembrane potentials waves: (1) ST elevated MI is simulated by decreasing the magnitudes of the transmembrane potentials wave by 48%^29^, and (2) the time instants of the transmembrane potentials wave was shortened to get increased T peak MI case. The amplitudes of the abnormal epicardial potentials are shown in Fig. 1c.

The current inverse study is tested on another heart model (all simulations were designed and executed in SCIRUN/BioPSE software^26^) and the models were imported from ECGSim software^25^ as source with the same diseased cases as shown in Fig. 1b. The model consisted of 585 discretized nodes with 1156 triangular surface mesh elements. The source sampling in this structure is more than the previous heart model. The reason for performing the inverse study on this test model is explained in the succeeding sections.

#### Transfer matrix

After modeling the structural and functional prior assumptions of the heart aligned with the torso, the spatial detector vector ${\textbf {m}}= ({\textbf {m}}_1,\, {\textbf {m}}_2,\,\ldots \, {\textbf {m}}_M)$ are placed in parallel to the thorax. The MCG detectors are collected from a set of $9 \times 9$ observation points ($M = 81$) with 3 cm intervals in between (available in ECGsim^25^). Now, a transfer matrix is designed to construct the spatial sensitivity between *Q* sources and *M* detectors. The generic field is recorded by placing unit current dipole vectors $\hat{{\textbf {a}}}_q$ at the nodes of the myocardium with the help of Biot-Savarts law. The magnetic field observed at sensor array (position vector in three dimensional Cartesian coordinates denoted by ${\textbf {r}}_m= ({\textbf {r}}_1, {\textbf {r}}_2, {\textbf {r}}_3, \ldots {\textbf {r}}_M)$) due to unit dipoles at ${\textbf {r}}'_q =({\textbf {r}}'_1, {\textbf {r}}'_2, {\textbf {r}}'_3, \ldots {\textbf {r}}'_Q)$ heart points are appended for all the sources to construct a lead field spatial tensor of dimension ${M\times Q\times 3}$:

1 $$\begin{aligned} {\textbf {L}}({\textbf {r}}_m,\langle {\textbf {r}}'_q,\hat{{\textbf {a}}}_q \rangle ) =\frac{\mu _0}{4\pi } \frac{{\textbf {r}}_m-{\textbf {r}}'_q}{\Vert {\textbf {r}}_m-{\textbf {r}}'_q\Vert ^3}\times \hat{{\textbf {a}}}_q \end{aligned}$$

The tensor ${\textbf {L}}({\textbf {r}}_m,\langle {\textbf {r}}'_q,\hat{{\textbf {a}}}_q \rangle )$ is expressed as:
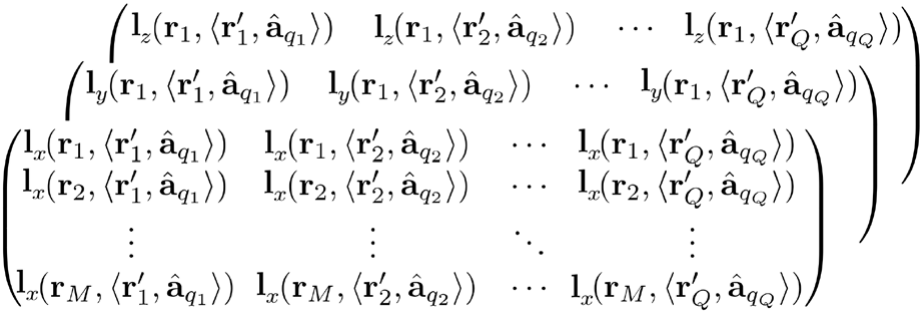


The elements of the constructed spatial transfer tensor ${\textbf {L}}({\textbf {r}}_m,\langle {\textbf {r}}'_q,\hat{{\textbf {a}}}_q \rangle ) =$ $[{\textbf {L}}_x({\textbf {r}}_m,\langle {\textbf {r}}'_q,\hat{{\textbf {a}}}_q \rangle ), {\textbf {L}}_y({\textbf {r}}_m,\langle {\textbf {r}}'_q,\hat{{\textbf {a}}}_q \rangle ),$ ${\textbf {L}}_z({\textbf {r}}_m,\langle {\textbf {r}}'_q,\hat{{\textbf {a}}}_q \rangle )]$ has the dimension $\mathbb {R}^{M\times Q\times 3}$; includes the locations and orientations of the sources, denoted as ${\textbf {L}}$ in further discussions.

#### Forward linear model

The forward magnetic fields (MCG) with dimensions $\mathbb {R}^{M\times t\times 3}$ are defined as an array of *M* detector coils, ${\textbf {B}}_f({\textbf {r}}_m,t) =[{\textbf {B}}_{fx}({\textbf {r}}_m,t),\, {\textbf {B}}_{fy}({\textbf {r}}_m,t),\, {\textbf {B}}_{fz}({\textbf {r}}_m,t) ]$ which are computed from the lead field matrix ${\textbf {L}}$ and prior scalar epicardial potential distributions $s(\langle {\textbf {r}}'_q,\hat{{\textbf {a}}}_q \rangle ,t) \in \mathbb {R}^{Q\times t}$. In further discussions throughout the article, ${\textbf {B}}_f({\textbf {r}}_m,t)$ and $s(\langle {\textbf {r}}'_q,\hat{{\textbf {a}}}_q \rangle ,t)$ are denoted as ${\textbf {B}}_f$ and *s*, respectively. The following equation represents the linear model to construct the forward magnetic field:

2 $$\begin{aligned} {\textbf {B}}_f={\textbf {L}}s \end{aligned}$$

The MCG waves are simulated for normal and abnormal cases and considered as true observed signals in the inverse problem. But, the usage of the same model to obtain the inverse solution leads to a problem called ‘inverse crime’  i.e., the results may yield best estimates which could be equivalent to the truth. One can overcome this problem by adding noise to the linear forward problem^30^. However, the actual modeling error of using the same models (unrealistic scenarios) in the inverse estimations is still a ‘crime’ and the results may end up providing over-estimated solutions^31,32^.

In our study, it has been attempted to solve the ‘inverse crime’. First the forward calculated magnetic field intensity (subjecting to uncertainties) is distorted by Gaussian noise with substantial amount of Signal-to-Noise Ratio (SNR) levels (ranging from 6 to 16 dB) and then the hidden potentials are determined. However as explained before, this does not solve the inverse crime so we further utilized the forward model of the different heart structure and the forward calculated magnetic field intensity obtained from the first source to estimate the unknowns.

**Figure 2:**
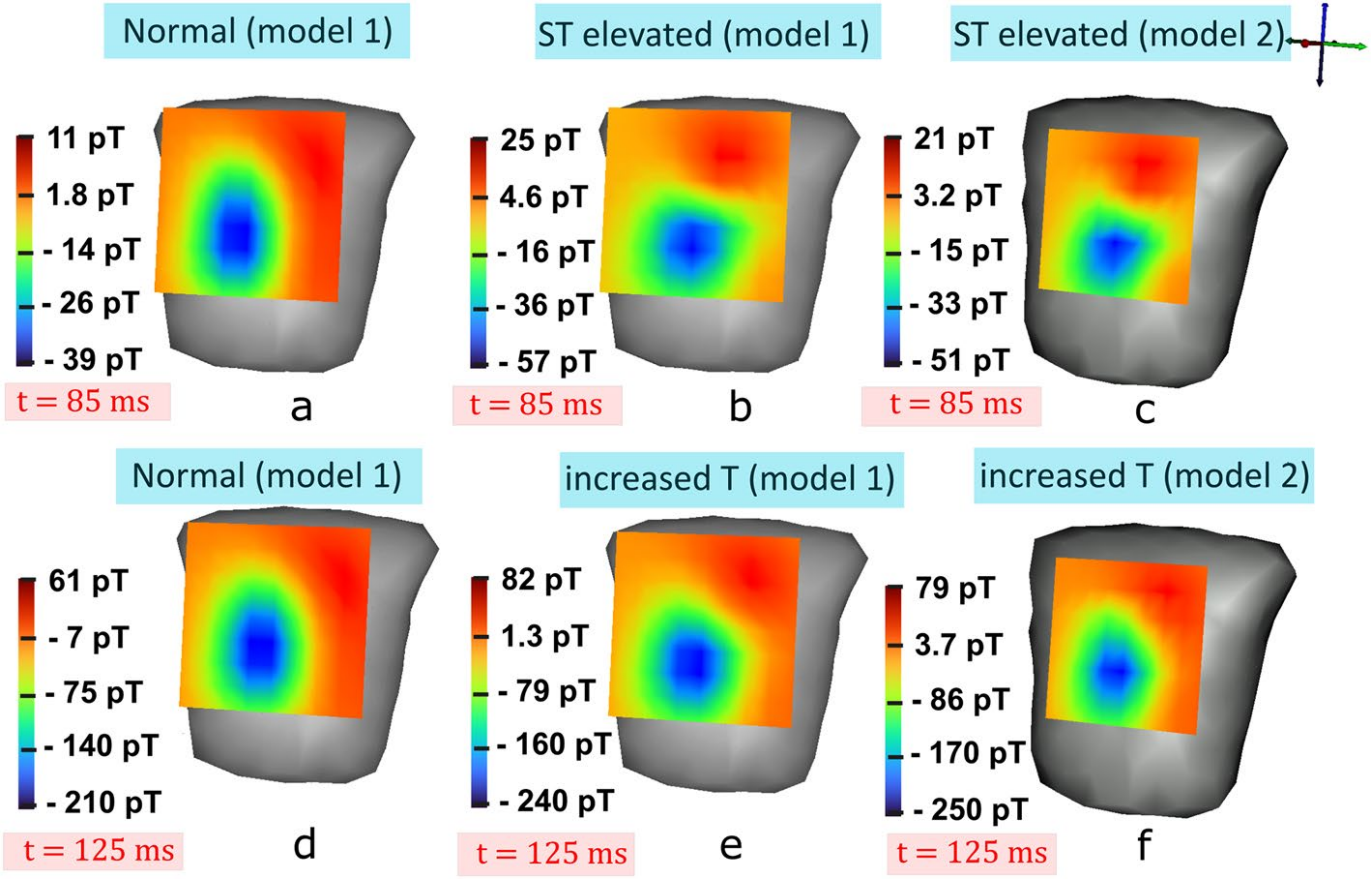
MCG signals mapped on to the detectors plane: (**a**) normal MCG map (model 1), and (**b**) ST elevated MCG of model 1 at 85 ms. (**c**) ST elevated MCG of model 2 at 85 ms. (**d**) Normal MCG (model 1) and (**e**) increased T maps captured of model 1 at 125 ms instant, and (**f**) increased T maps of model 2 captured at 125 ms instant (dark/red: maximum magnetic field intensities, light/blue: minimum magnetic field intensities).

Figure 2a,b represent the MCG maps at mid ST instants of normal and ST elevated MI case from the first heart model, respectively. Similarly, MCG fields at increased T peak maps of normal and abnormal of model 1 are illustrated in Fig. 2d,e, respectively. The forward calculated magnetic field intensity from the second source model (shown in Fig. 1b) are mapped in Fig. 2c,f depicting the ST elevated and increased T instants, respectively. The variations in the amplitudes of the field intensities can be visualized in the maps.

The linear forward field (${\textbf {B}}_f$) representing the cardiac cycle from $t = 1$s to $t = T$s, subjected to noise ${\textbf {n}}({\textbf {r}}_m,t)$ denoted as ${\textbf {n}}$ is formulated to get ${\textbf {B}}_{fn}$ and is expressed as:

3 $$\begin{aligned} {\textbf {B}}_{fn} ={\textbf {B}}_f+{\textbf {n}} \end{aligned}$$

The noise ${\textbf {n}}$ of time frame *T*s is derived from the SNR dB with signal power $\left( P_s = \frac{1}{T}\int _{t=1}^T\vert {\textbf {B}}_f\vert ^2 dt\right)$ and noise power $\left( P_n = \frac{1}{T}\int _{t=1}^T\vert {\textbf {n}}\vert ^2 dt\right)$ is given by:

4 $$\begin{aligned} SNR (dB) = 10\log _{10}\left( \frac{P_s}{P_n}\right) \end{aligned}$$

Since the noise power is not known, the parameter of a specific noise amplitude is formed using the above equation that reduces to

5 $$\begin{aligned} P_n=\sqrt{\frac{P_s}{10^{\frac {SNR}{10}}}} \end{aligned}$$

for a range of SNR from 6 to 16 dB. The obtained noise power amplitude $P_n$ is then multiplied with the random noise sequence of the length similar to the signal’s time points.

### Inverse problem

The ill-posed inverse problems are solved after establishing the forward model. In this paper, Deterministic and Probabilistic approaches for inverse problems are implemented and their efficiencies in noiseless and noisy conditions compared.

#### Deterministic approach: Tikhonov regularization

The unknown source activations are estimated with the help of transfer matrix ${\textbf {L}}$ and from known observations ${\textbf {B}}({\textbf {r}}_m,t)$ denoted as ${\textbf {B}}$ using squared error function^14^. The general cost function to be minimized is computed using:

6 $$\begin{aligned} \mathcal {F}(\cdot )=\Vert {\textbf {B}} -{\textbf {B}}_{fn}\Vert ^2 \end{aligned}$$

Since the system is under-determined ($M<Q$), it causes over-fitting in the function. In order to overcome this problem, a penalty term called solution norm (L2 norm) is introduced that contains squared magnitude of the epicardial source weights. The standard Tikhonov (L2 norm) regularization implemented in the study, is expressed as:

7 $$\begin{aligned} \mathcal {F}(\cdot )=\Vert {\textbf {B}}-({\textbf {L}} s +{\textbf {n}}) \Vert ^2+\lambda _R\Vert s \Vert ^2 \end{aligned}$$

where $\lambda _R$ is the regularization parameter which controls and helps in fitting the sources by minimizing the cost function.

### Hierarchical Bayesian framework

In the Bayesian estimation, the magnitudes of unknown source activities are estimated, which are priorly assumed to be random with known prior distributions. The joint probability density function (pdf) of the magnetic field data ${\textbf {B}}$ and source activities *s* are assumed to be normally distributed. In this, *p*(*s*) is the prior distribution of *s* and $p({\textbf {B}}\mid s)$ is the likelihood function of MCG conditioned on the sources *s*. The posterior distribution to estimate the activities is written as:

8 $$\begin{aligned} p({s}\mid {\textbf {B}})=\frac{p({\textbf {B}}\mid s)\cdot p(s)}{p({\textbf {B}})} \end{aligned}$$

where $p({\textbf {B}})$ in the denominator is the normalizing factor of observed signals.

The conditional probability of the complete MCG cardiac cycle from *i*th detector ${\textbf {B}}_{it} = {\textbf {B}}({\textbf {r}}_m={\textbf {r}}_i,t)$ with time points ($t= 1,\ldots ,T$) given the whole latents of the epicardial potentials $s_{qt}=s(\langle {\textbf {r}}_q',\hat{{\textbf {a}}}_q\rangle ,t)$ considering the lead field (${\textbf {L}}$) is given by,

9 $$\begin{aligned} p({\textbf {B}}\mid s)= \prod _{t = 1}^{T} p({\textbf {B}}_{it}\mid s_{qt}) \sim \prod _{t = 1}^{T} \mathcal {N}({\textbf {B}}_{it}\mid {\textbf {L}}_{iq} s_{qt}, \Lambda ^{-1}) \end{aligned}$$

We assume the noise ${\textbf {n}}$ (Eq. 3) is Gaussian with zero mean and is *i.i.d* across time expressed as

10 $$\begin{aligned} {\textbf {n}} \sim \mathcal {N}({\textbf {n}}\mid 0, \Lambda ^{-1}) \end{aligned}$$

Here, $\Lambda$ is a diagonal precision matrix in which the diagonal entries are equal to the inverse of the noise variances for the corresponding observed MCG data.

The forward likelihood over the MCG at *M* detectors in an instant is defined as:

11 $$\begin{aligned} p({\textbf {B}}\mid s) \propto \frac{\vert \Lambda \vert ^{\frac {1}{2}}}{2\pi ^{\frac {M}{2}}} \exp \left[ -\frac{ \Lambda }{2} \Vert {\textbf {B}}-({\textbf {L}} s+{\textbf {n}})\Vert ^2\right] \end{aligned}$$

Similarly, the prior distribution of the epicardials $s_{jt}$ for a *j*th source is assumed to be Gaussian and *i.i.d* across time and the model for the entire time series of $s_{jt}$ ($t=1,\ldots T$) is given by

12 $$\begin{aligned} p(s)=\prod _{t=1}^{T}p(s_{jt})\sim \prod _{t=1}^{T} \mathcal {N}(s_{jt}\mid 0, \Phi ^{-1}) \end{aligned}$$

Here, $\Phi$ is the precision or covariance matrix which contains the inverse of the source variances ($\sigma _s^2$).

The explicit form of the Gaussian distribution of the epicardial priors at *Q* sources in a specific instant is given by

13 $$\begin{aligned} p(s) \propto \frac{\vert \Phi \vert ^{\frac {1}{2}}}{2\pi ^{\frac {Q}{2}}} \exp \left[ -\frac{ \Phi }{2} \Vert s\Vert ^2\right] \end{aligned}$$

Two scalar parameters $\alpha$ and $\beta$ called hyperparameters are introduced to control the distribution of the parameter *s*. The hyperparameters supporting the precision matrices are assumed as $\Phi =\alpha I$ and $\Lambda = \beta I$ for prior source and noise covariances, respectively.

These precision hyperparameters decide the variance estimates of the posterior distribution. The posterior distribution of the epicardial weights (Gaussian) for any source at ${\textbf {r}}'_q$ is formed as

14 $$\begin{aligned} p(\hat{s}_{jt}\mid {\textbf {B}}_{it}, s_{jt},\alpha ,\beta )\sim \prod _{t=1}^{T} \mathcal {N}(s_{jt}\mid \hat{s}_{jt}, \Sigma ^{-1}) \end{aligned}$$

where the mean and variance are given by

$$\begin{aligned} \hat{s}_{jt}= \beta \Sigma ^{-1}{\textbf {L}}^\top {\textbf {B}}_{it} \end{aligned}$$

$$\begin{aligned} \Sigma ^{-1}=\alpha I+\beta {\textbf {L}}^\top {\textbf {L}} \end{aligned}$$

The values of the hyperparameters are unknown and are derived from the data. This can be done by introducing prior distributions over the hyperparameters $\alpha$ and $\beta$, and predicting the posteriors by marginalizing with respect to these hyperparameters and epicardial weights *s*.

We employed the method of evidence approximation, since the complete marginalization over *s*, $\alpha$, and $\beta$ by integrating analytically is not tractable^21,30^.

If the hyperpriors are modeled over $\alpha$ and $\beta$, the predictive distribution by marginalizing over *s*, $\alpha$, and $\beta$ gives

15 $$\begin{aligned} p(s\mid {\textbf {B}}){} & {} = \int \int p(s,\alpha , \beta \mid {\textbf {B}}) d\alpha d\beta \nonumber \\{} & {} = \int \int p(s\mid \alpha , \beta , {\textbf {B}}) p(\alpha ,\beta \mid {\textbf {B}}) d\alpha d\beta \end{aligned}$$

If the hyperposteriors $p(\alpha ,\beta \mid {\textbf {B}})$ are most probable around the values $\hat{\alpha }$ and $\hat{\beta }$, the posterior distribution of epicardials is computed by marginalizing over *s* with $\hat{\alpha }$ and $\hat{\beta }$. From Bayes’ theorem, hyperposteriors are expressed as

16 $$\begin{aligned} p(\alpha ,\beta \mid {\textbf {B}}) \propto p({\textbf {B}}\mid \alpha , \beta ) p(\alpha , \beta ) \end{aligned}$$

Thus, by maximizing the $p({\textbf {B}}\mid \alpha , \beta )$ provides the evidence for $\alpha$ and $\beta$.

The marginal likelihood of the MCG data $p({\textbf {B}}\mid \alpha , \beta )$ is obtained by integrating over the sources *s*,

17 $$\begin{aligned} p({\textbf {B}}\mid \alpha , \beta )=\int p({\textbf {B}}\mid s, \beta ) p(s\mid \alpha ) ds \end{aligned}$$

From Eqs. (9) and (12), the evidence function is written in the form

18 $$\begin{aligned} p({\textbf {B}}\mid \alpha , \beta )=\left( \frac{\beta }{2\pi }\right) ^{\frac{M}{2}} \left( \frac{\alpha }{2\pi }\right) ^{\frac{Q}{2}}\int \exp \{-E(s)\} ds \end{aligned}$$

where

19 $$\begin{aligned} E(s)= \frac{\beta }{2}\Vert {\textbf {B}}-({\textbf {L}}s+{\textbf {n}})\Vert ^2+\frac{\alpha }{2}\Vert s \Vert ^2 \end{aligned}$$

The expression $\int \exp \{-E(s)\} ds$ cannot be obtained analytically and practical solution of the same over *s* is larger. To simplify this, Taylor expansion of *E*(*s*) is considered around the minimum value by retaining up to the second order^23^. The expansion of *E*(*s*) around its minimum value is given by

20 $$\begin{aligned} E(s)= E_{s_{MP}}+\frac{1}{1!} f'(E_{s_{MP}}) (s-s_{MP}) + \frac{1}{2!} (s-s_{MP})^\top f''(E_{s_{MP}})(s-s_{MP}) \end{aligned}$$

where $s_{MP}$ denotes the most probable solution (epicardial potentials). The second term in the expansion is the minimum value and can be discarded or equated to zero. The second order derivative function is

$$\begin{aligned} f''(E_{s_{MP}})= \beta {\textbf {L}}^\top {\textbf {L}}+\alpha I \end{aligned}$$

and

$$\begin{aligned} E_{s_{MP}}= \frac{\beta }{2}\Vert {\textbf {B}}-({\textbf {L}}s_{MP}+{\textbf {n}})\Vert ^2+\frac{\alpha }{2}\Vert s_{MP} \Vert ^2 \end{aligned}$$

By differentiating the log evidence of Eq. (18) with respect to $\alpha$, we get,

21 $$\begin{aligned} \hat{\alpha }=\frac{\gamma }{2\Vert s_{MP} \Vert ^2} \end{aligned}$$

where $\gamma =\frac{\lambda _i}{\lambda _i+\alpha }$ & $\lambda _i(i=1,\ldots ,Q)$ are the eigen values of $\beta {\textbf {L}}^\top {\textbf {L}}$.

Similarly, by maximizing the log evidence with respect to $\beta$, results in

22 $$\begin{aligned} \beta = \frac{M-\gamma }{2\Vert {\textbf {B}}-({\textbf {L}}s_{MP}+{\textbf {n}})\Vert ^2} \end{aligned}$$

The Algorithm 1 explains the source estimation procedure by updating the hyper-parameters in iterative manner until it reaches to a convergence criteria. 
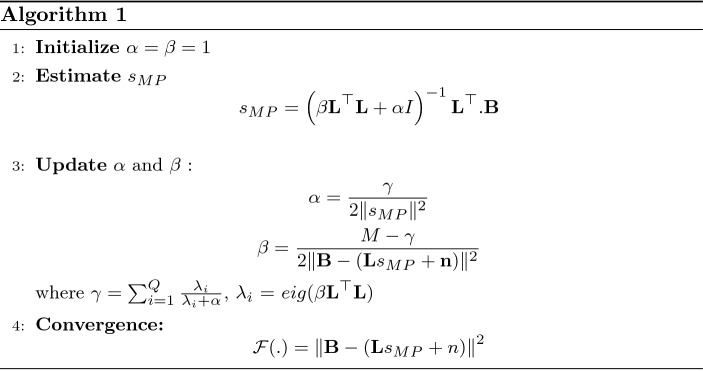


### Variational Bayesian linear regression

The Variational Bayesian linear regression method is a probabilistic algorithm previously used in solving other applications as seen in^33^. So in this paper, this method has been explored for the first time to localize the sources from MCG signals.

#### Bayesian model

The linear model explained in the forward model (Eq. 2) that assumes linear relation between *Q* dimensional *s* sources and *M* dimensional MCG observations with independent noise at the detectors is considered in the inverse procedure. The magnetic field intensity likelihood of the observations ${\textbf {B}}$ (similar to the previous approach) is assumed with constant-variance Gaussian noise distribution $\Lambda =\beta I$^20^, defined as:

23 $$\begin{aligned} p({\textbf {B}}\mid {\textbf {L}},s,\beta ) \sim \mathcal {N}({\textbf {B}}\mid {\textbf {L}} s, \Lambda ^{-1}) \end{aligned}$$

**Figure 3:**
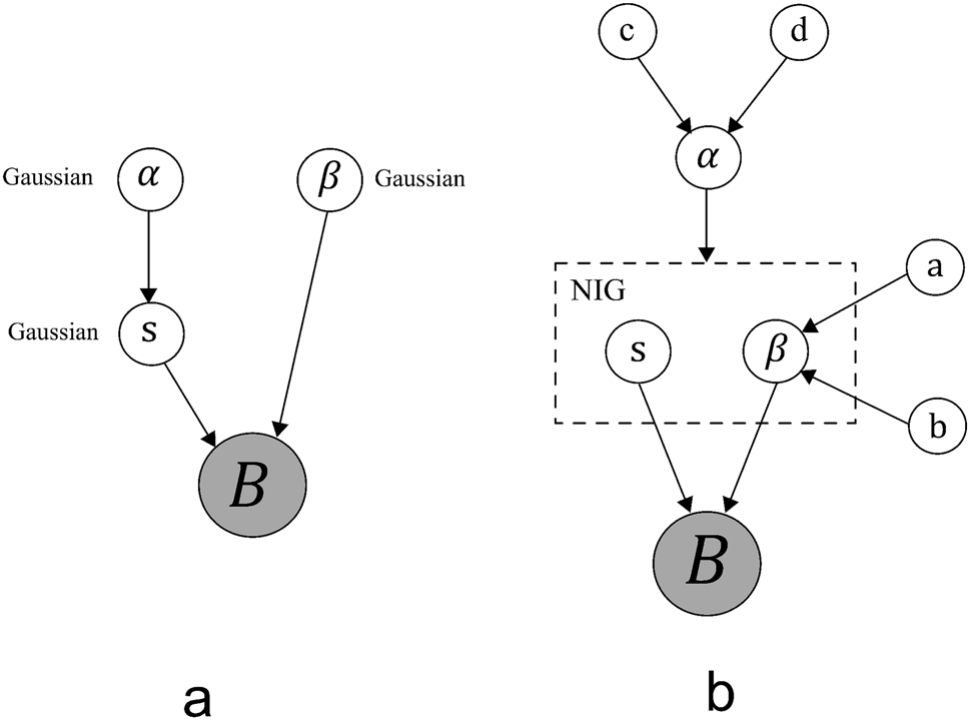
Graphical models representing the relationship between the variables: (**a**) Bayesian model with Gaussian priors, (**b**) Variational Bayesian linear model with Normal inverse-gamma priors.

The graphical representation of Bayesian models is illustrated in Fig. 3a,b to understand the relationships between variables.

#### Priors and hyper-priors

In the previous section (simplistic Bayesian approach), the prior source weights in the linear model were assumed to be Gaussian distribution. Due to this stationary prior, it is possible to estimate marginal likelihood and obtain posteriors^19^. It is also important to extract the sparser characteristics of the unknown signals such as region of abnormal spread estimations in the epicardial potentials that appear to be smoother solutions in stationary prior. To overcome this problem, a non-stationary conjugate normal inverse-gamma distribution is assumed on the prior sources *s* and variance $\Lambda =\beta I$, parametrized by hyper prior $\Phi =\alpha I$ graphically represented in Fig. 3. The prior source distribution is modeled as:

24 $$\begin{aligned} p(s,\beta \mid \alpha ) \sim \mathcal {N}({s\mid 0,(\beta \alpha )^{-1}I}) \cdot \; Gam(\beta \mid a_0,b_0) \end{aligned}$$

In this prior, $\beta$ acts as inverse variance on *s* with zero-mean. Due to imposition of Gamma on $\beta$, $\beta ^{-1}$ is defined as the inverse-gamma function with shape $a_0$ and scale $b_0$ and is expressed as:

25 $$\begin{aligned} p(s,\beta \mid \alpha )=\left( \dfrac{\alpha }{2\pi }\right) ^{\frac {Q}{2}} \dfrac{b_0^{a_0}}{\Gamma (a_0)} \beta ^{\frac {Q}{2}+a_0-1}\exp \left( -\eta \right) \end{aligned}$$

where $$\eta =\dfrac{\beta }{2}(\alpha s^\top s)+2b_0$$

The convergence criteria of the algorithm 1 is evaluated by checking the change in updates of hyperparameters. This is done by setting a stop threshold $\epsilon = 10^{-3}$ value to track the difference between the hyperparameter updates. Then the algorithm is constrained to stop at iteration *j* if $P(j)<\epsilon$ where:

26 $$\begin{aligned} P(j)=\max \{\vert \alpha (j)-\alpha (j-1)\vert , \vert \beta (j)-\beta (j-1)\vert \} \end{aligned}$$

#### Variational inference

The final step is to estimate the cardiac sources in terms of variational posteriors termed as $p(s,\beta ,\alpha \mid {\textbf {B}})$. Since it is difficult to obtain the closed form of posteriors, a distribution $g(s,\beta ,\alpha )$ is introduced in the model to solve it as variational approximation. With the help of Kullback-Leibler (KL) divergence, the difference between $g(s,\beta ,\alpha )$ and posterior $p(s,\beta ,\alpha \mid {\textbf {B}})$ is minimized, defined as:

27 $$\begin{aligned} KL(g\mid \mid p)=\int _{s,\beta ,\alpha }g\cdot ln \left( \frac{g }{p}\right) ds d\beta d\alpha \end{aligned}$$

where $g=g(s,\beta ,\alpha )$ and $p=p(s,\beta ,\alpha \mid {\textbf {B}})$. The distribution $g(\cdot )$ is computed by minimizing the KL divergence:

28 $$\begin{aligned} g = \arg min_{g(s,\beta ) g(\alpha )} KL(g\Vert p) \end{aligned}$$

The main idea is to find the distribution *g* that is closer to the distribution *p*. The divergence in Eq. (27) reduces to:

29 $$\begin{aligned} KL(g\Vert p)=-\mathcal {L}(g)+ln \;p({\textbf {B}}) \end{aligned}$$

Now, with the help of known $ln\; p({\textbf {B}})$, the variational posteriors $g(s,\beta )$ and $g(\alpha )$ are estimated by maximizing the variational lower bound $\mathcal {L}(g)$^23^ (equivalent to minimizing the KL divergence function in Eq. (28)).

The variational posteriors for *s* and $\beta$, with fixed $g(\alpha )$ is given by:

30 $$\begin{aligned} ln \;g(s,\beta ) = ln\; p({\textbf {B}}\mid {\textbf {L}},s,\beta )+E_\alpha \left( ln\; p(s,\beta \mid \alpha )\right) + constant \end{aligned}$$

The posterior on sources with variance $g(s,\beta )$ reduces to:

31 $$\begin{aligned} ln\; g(s,\beta ) = ln \;\left( \mathcal {N}(s\mid \hat{s},\beta ^{-1}\hat{C}) \right) \cdot \; Gam(\beta \mid \hat{a},\hat{b}) \end{aligned}$$

where *C* is the covariance matrix of sources, *a* and *b* are the shaping and scaling parameters influencing the hypers $\beta$. Similarly, by differentiating the lower bound $\mathcal {L}(g)$ with respect to $\alpha$ by keeping *s* and $\beta$ as constant:

32 $$\begin{aligned} ln\; g(\alpha )=E_{s,\beta }\left( ln\; p(s,\beta \mid \alpha )\right) +ln\; p(\alpha ) + constant \end{aligned}$$

The variational posteriors on $\alpha$ reduces to:

33 $$\begin{aligned} ln\; g(\alpha )=ln\;Gam(\alpha \mid \hat{c},\hat{d}) \end{aligned}$$

Algorithm 2 explains the reconstruction of epicardial potentials from MCG observation using Variational Bayesian linear regression technique. 
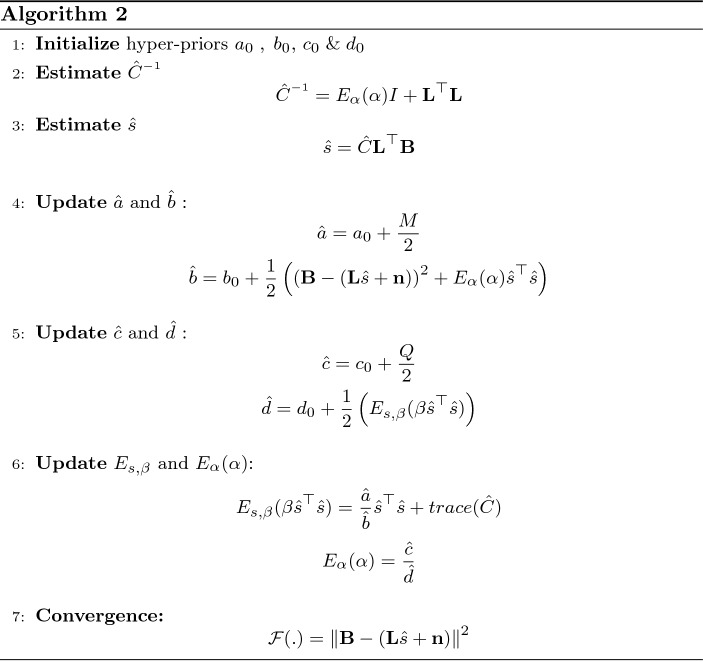


 The hyperparameter update is terminated when the variational lower bound $\mathcal {L}(g)$ remains unchanged for more than 0.001% between the two successive iterations.

## Results and discussion

**Figure 4:**
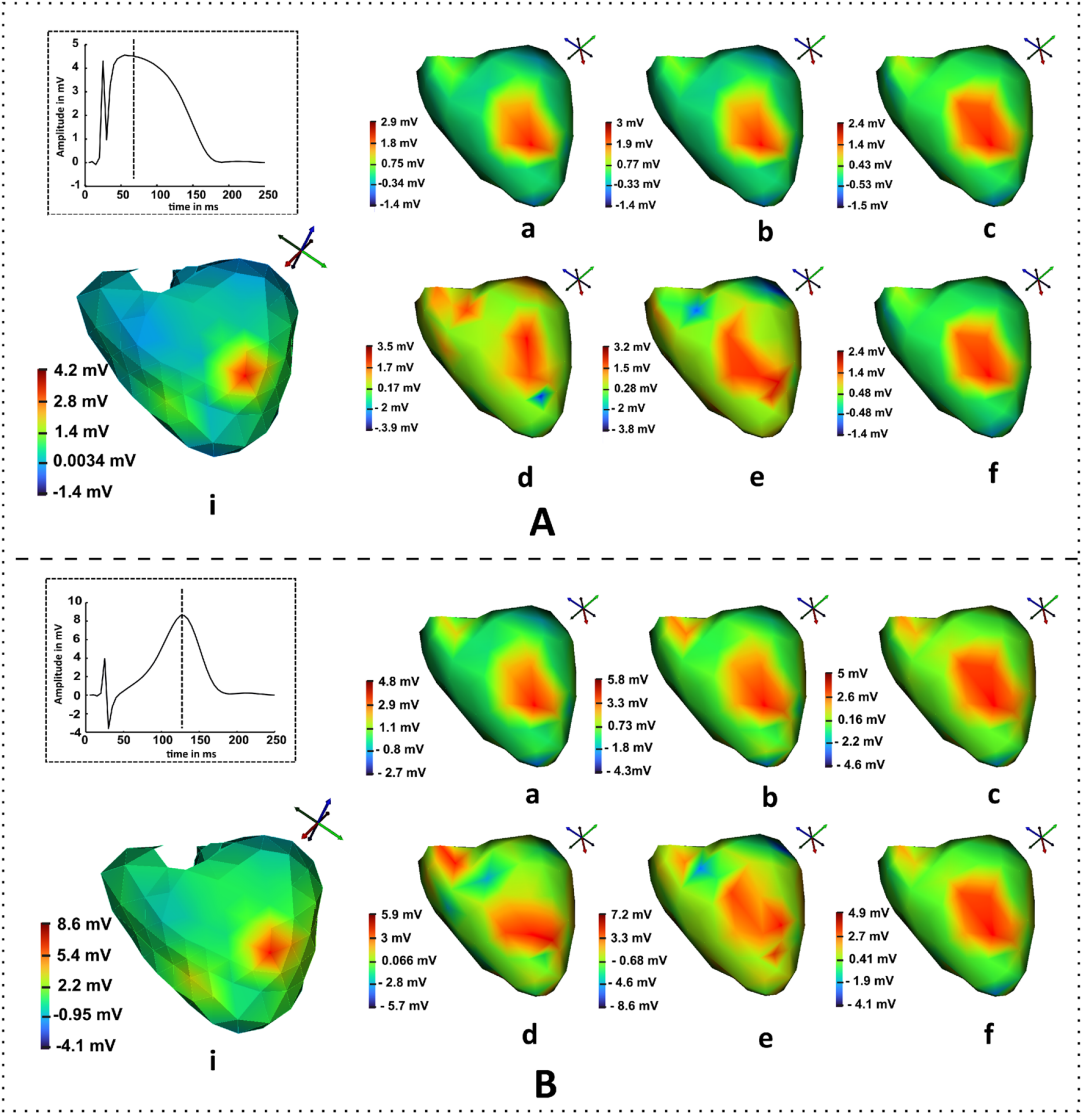
Results of epicardial map estimations obtained from inverse algorithms at the desired time instant: (**A.i**) reference map of model 1 at t = 85 ms (inset: epicardial of the diseased node with time point t = 85 ms shown as a dotted vertical line ) Inverse solutions solved on model 2 at noise-free condition from (**a**) Tikhonov, (**b**) Bayesian and, (**c**) VBLR methods, whereas the second row shows the results obtained for (**d**) Tikhonov, (**e**) Bayesian and, (**f**) VBLR in 8 dB SNR. Similarly, the results of the increased T peak solutions at t = 125 ms are represented in (**B**).

In this section, the performance of algorithms 1 and 2 for epicardial source reconstructions are compared with that of deterministic method in noise-free and noisy conditions. The inverse algorithms are computed in MATLAB and the results are visualized in SCIRun software^25,30^. The true epicardial maps were generated using the geometry of model 1 (mappings at t= 85ms and t= 125 ms shown in Fig. 4A.i,B.i, respectively) while the transfer matrix generated from the geometry of model 2 was used to solve the inverse problem. Due to this reason the inverse crime was avoided however it led to some significant modeling errors. The results obtained from the Tikhonov approach in noiseless condition ranged from − 1.4 to 2.9 mV; where the region of spread showed a similar kind of ruptured information as that of true potentials (shown in Fig. 4A.a). The magnitudes of the spread regions of the Bayesian inference (Fig. 4A.b) yielded a reasonable amount of improvement in the results than the Tikhonov method and the solutions ranged from − 1.4 to 3 mV in noise-free case. The main study was to visualize and evaluate the results for uncertainty conditions.

This is performed by adding a noise of 8 dB SNR to the forward magnetic field intensity and solving the inverse problems. It can be observed from the Fig. 4A.d that the Tikhonov method in the noisy cases produced the contorted reconstruction spread at the diseased node. Figure 4A.e represents the Bayesian estimation results where the distributed area surrounding the diseased node was noticeable with magnitude ranges [− 3.8 3.2] mV. The VBLR approach performed well in both noise-free and noisy conditions evident from the maps shown in Fig. 4A.c,A.f, respectively. Similarly, Fig. 4B.a–c describe the estimations solved by Tikhonov, Bayesian inference, and VBLR methods, respectively at t = 125 ms. Also, the inverse solutions obtained at this instant from Tikhonov, Bayesian inference, and VBLR techniques due to the impact of noises in the forward field are shown in Fig. 4B.d–f, respectively.

**Figure 5:**
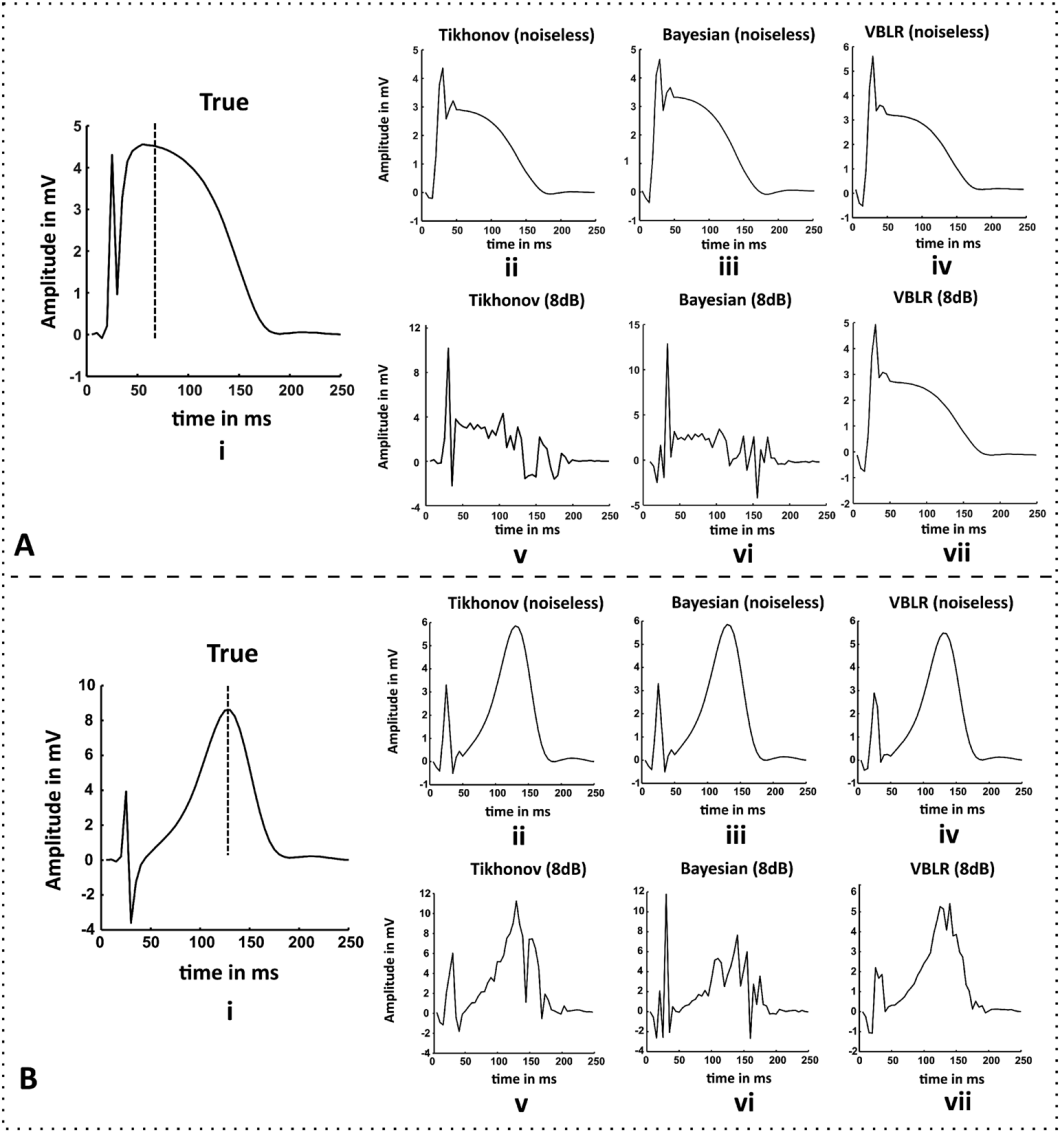
(**A.i**) True epicardial potentials of ST elevated MI (model 1) at the diseased node (vertical dotted line: time stamp at t = 85ms), (**B.i**) True heart potentials of T elevated case from model 1 (vertical dotted line: time stamp at t = 125 ms). Results of the epicardial estimations obtained from inverse algorithms on the model 2 with respect to time for both the cases (**A**,**B**): (**ii**) standard Tikhonov regularization, (**iii**) Bayesian modeling with Gaussian priors and (**iv**) variational Bayesian inference in noise free conditions, and reconstructions from (**v**) Tikhonov regularization, (**vi**) Bayesian modeling, and (**vii**) Variational Bayesian inference method for 8 dB SNR, respectively.

Figure 5 depicts the time series reconstructions of the heart surface potentials at the desired node index. The potentials activated in the model 1 (shown in Fig. 5A.i,B.i) are considered as the reference truth values for ST elevated and increased T cases, respectively. As compared to the true values, the amplitudes reconstructed by the VBLR were less than the Bayesian solutions (visually—for elevated ST case shown in Fig. 5A.iii,iv); but the temporal RMSEs of the VBLR method outperformed the Bayesian method in both noisy and noise-free conditions which are later discussed in Table 2.

It can be observed that the Tikhonov approach attempted to produce good solution as shown in Fig. 5A.v,B.v visually, but the amplitudes are not satisfactory which still showed the elements of noise present in the estimated results. This shows that uncertainties in the forward magnetic field intensity are less handled by deterministic methods. The solution of the Bayesian inference (Fig. 5A.vi,B.vi) traced out better estimates than the Tikhonov method. The temporal reconstruction of the potentials showed remarkably better results in VBLR method even under 8 dB SNR (Fig. 5A.vii,B.vii) than the other methods.

### Choice of regularization parameter

**Figure 6:**
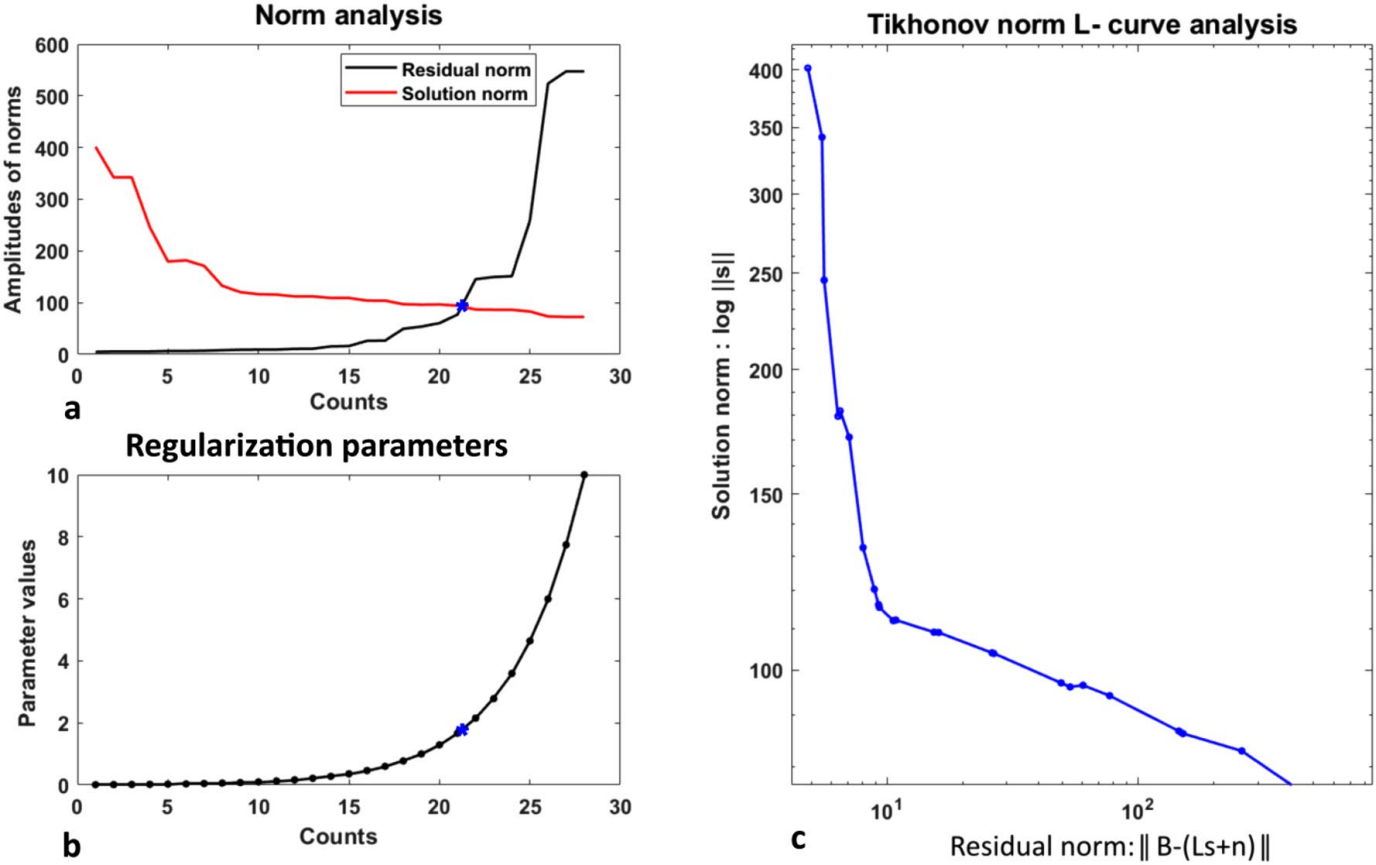
Selection of an optimum regularization parameter using L-curve criterion. (**a**) Plots of the residual norms and the solution norms, (**b**) Range of the $\lambda$ values used in the algorithm (asterisks in a and b represent the trade-off optimal point), (**c**) L-curve optimization plot for the Tikhonov solution at t = 85 ms (ST elevated case).

In the regularization theory, the selection of an optimum value is a tricky and challenging task that provides a good solution of fit. L-curve is the commonly used approach to choose an optimum regularization parameter in the Tikhonov estimation. The parametric L-curve graph is the $\log -\log$ plot of the regularized solution norm ($\Vert s \Vert$) versus the norm of the corresponding residuals ($\Vert {\textbf {B}}-({\textbf {L}}s+{\textbf {n}})\Vert$) shown for ST elevated solution in Fig. 6. As the regularization parameter is varied, the graphical tool displays the trade-off occurring between the regularized solution and its fit to the observed data. In the work, the regularization parameter was varied from $\lambda =1E-10$ to $\lambda =10$ with 100 linearly spaced vectors in between them. The optimal regularizer can be found at a corner of the curve isolating the horizontal and vertical lines in the $\log -\log$ scale.

The vertical part of the curve in Fig. 6c corresponds to the solutions with small $\lambda$ values. As the $\lambda$ values are tuned to the small values, the solution norm starts increasing, thereby decreasing the norm of the residuals as shown in the Fig.  6a,b (for $\lambda = 0.01$, the values of $\Vert s\Vert = 401.582$, and the residual norm found was $\Vert {\textbf {B}}-({\textbf {L}}s+{\textbf {n}})\Vert = 4.8466$).

The solution norm decreases slightly for large $\lambda$ values causing the solution to over regularize (and increases the residual norm); corresponds to the horizontal part of the L-curve. The corner in Fig. 6c is considered as an optimum value $\lambda = 1.6681$ that yields a good solution fit for the latent t = 85 ms (mid ST elevated point).

The inverse algorithms are tested by applying different levels of noise (SNR ranging from 6 to 16 dB) to the MCG system and Root Mean Square Error (RMSE) between the true (assumed) and estimated potentials are computed for quantitative evaluations and is defined as:

34 $$\begin{aligned} sRMSE = \sqrt{\frac{\sum _{q=1}^{Q} \left( s_q-\hat{s}_q\right) ^2}{Q}} \end{aligned}$$

**Figure 7:**
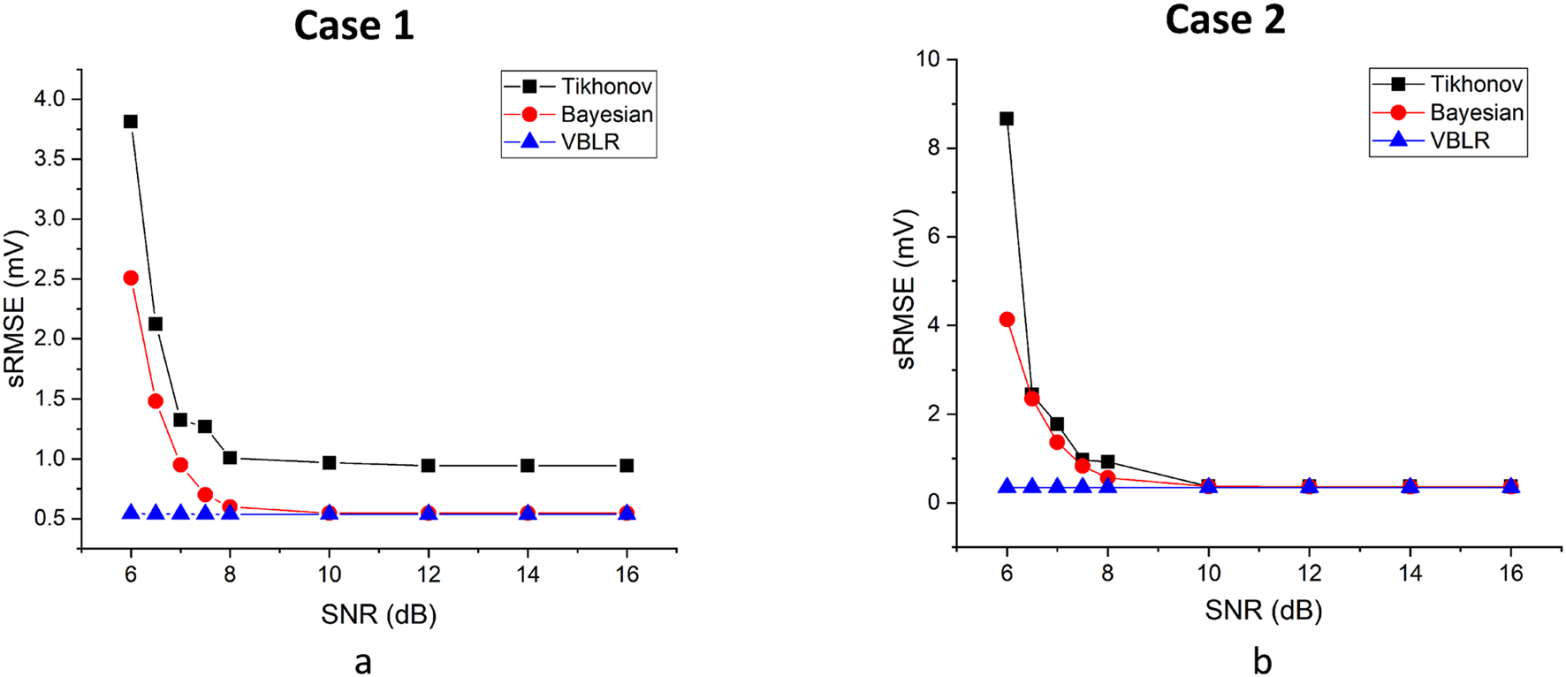
Performance evaluation of algorithms using $sRMSE$ compared under different levels of noise in forward magnetic field ((**a**) Case 1: ST elevated at t= 85 ms, (**b**) Case 2: increased T at t = 125 ms).

where $s_q$ denotes the assumed epicardial potentials for two different heart models with geometrical nodes $Q_1$ and $Q_2$ , and $\hat{s}_q$ represents the estimated potentials at the same spatial points $Q_1$ and $Q_2$.

Figure 7a,b show the plot of SNR versus $sRMSE$ applied on deterministic and Bayesian algorithms for ST elevated MI and increased T cases, respectively. The $sRMSE$ values obtained in the worst case scenarios (6 dB) for the Tikhonov method at t = 85 ms was 3.8 mV and at t = 125 ms was 8.66 mV. The Bayesian inference performed well in estimating the sources in 6 dB case by reaching to an $sRMSE$ value of 2.50 mV. Both Tikhonov and Bayesian results yielded constant $sRMSE$s of 0.94 and 0.546, respectively after the noise-levels of 12 dB.

**Figure 8:**
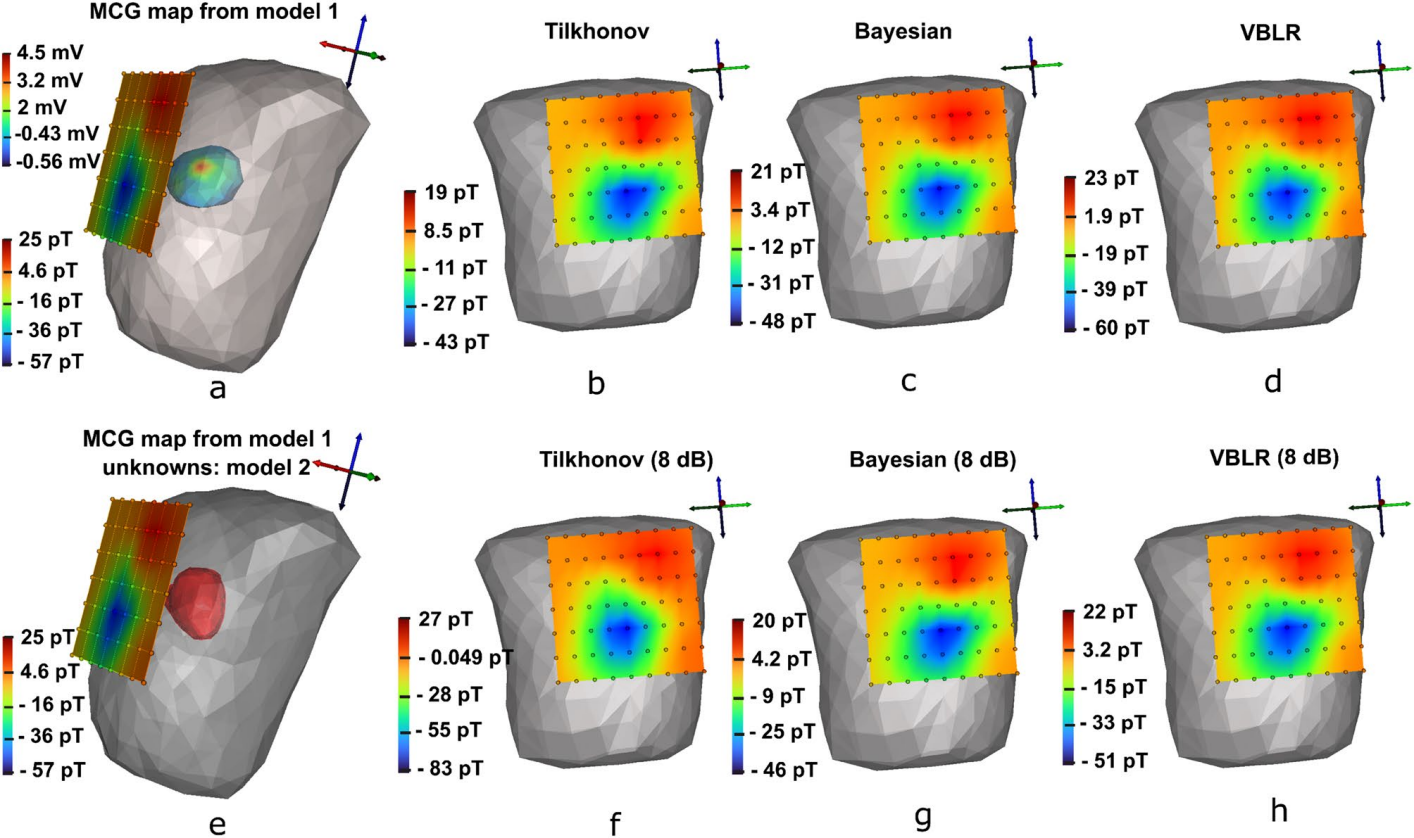
Reconstruction of MCG maps from the estimated epicardials of the model 2 at t = 85 ms, (**a**) true forward magnetic field intensity (pico Tesla—pT) from the epicardial potentials (milli Volts—mV) of the heart model 1 (shown inside the thorax), (**e**) utilization of MCG forward calculated magnetic field intensity from model 1 but the objective is to seek the unknown potentials of model 2 at t = 85 ms, (**b**–**d**) reconstructions of solutions estimated on model 2 for Tikhonov, Bayesian and, VBLR in noise-free environments on model 2, (**f**–**h**) reconstructions of solutions from model 2 for Tikhonov, Bayesian and, VBLR subjected to 8 dB SNR. (Dark/red: maximum magnetic field intensities, light/blue: minimum magnetic field intensities).

It can be seen that both the Bayesian and Tikhonov methods take time to adjust themselves from noisy to less noisy conditions however VBLR always maintains similar values and hence it can be said that it works best even in noisy conditions. The VBLR method thus outperforms the other methods by showing good $sRMSE$s of 0.5428 mV even in worst cases from 6 to 8 dB and reached to a constant $sRMSE$s of 0.538 mV after 8 dB.

Similarly, the VBLR showed good estimations in the second case at t = 125 ms, with $sRMSE$s of 0.347 mV for all the noise levels whereas the hierarchical Bayesian inference provided negligibly better solutions than the Tikhonov approach from 6 to 8 dB and reached constant $sRMSE$s of 0.3672 mV after 8 dB in both the methods. Thus we can conclude that the VBLR method provides far better estimates of $sRMSE$ values than Tikhonov and Bayesian methods in noisy cases.

Another metric called Correlation Co-efficient (CC) used in the study is tabulated in Table 1. The results of CC obtained for ST elevated case reached to 0.14 which is a very low value and it got improved by 1.7 times in the Bayesian approaches under noisy conditions.

Along with this, the temporal RMSEs are evaluated to check the performances of the estimated epicardials with respect time formulated as:

35 $$\begin{aligned} tRMSE = \sqrt{\frac{\sum _{t=1}^{T} \left( s_t-\hat{s}_t\right) ^2}{T}} \end{aligned}$$

As it can be observed from Table 1, the $tRMSE$ values of the VBLR method reached to 1.807 mV for ST elevated case at 6 dB SNR that can be considered as a good estimate, whereas the $tRMSE$s of the Tikhonov and Bayesian yielded 22.65 mV and 21.52 mV at noisy conditions.

**Table 1:** Evaluation of the inverse algorithms in terms of temporal RMSEs in mV and CC.

Condition	Cases	Tikhonov		Bayesian		VBLR	
		$tRMSE$	CC	$tRMSE$	CC	$tRMSE$	CC
Noise-free	Elevated ST	1.1577	0.7840	1.0091	0.8343	1.2627	0.8169
	Increased T	1.6185	0.6872	1.414	0.7966	1.0917	0.7817
Noisy: 6 dB SNR	elevated ST	22.6499	0.1416	21.5155	0.3832	1.2627	0.3873
	Increased T	23.3212	0.2022	21.7024	0.3347	1.8068	0.3348

Figure 8a represents the true MCG (range [− 57 25] pT) mapped at 85 ms for ST elevated MI case with epicardial potential spread of abnormal node (model 1 range [− 0.56 4.5] mV ) inside the torso. Now, the observed MCG is considered as the test data to solve the inverse problem for model 2 (shown in Fig.  8e). The results of the epicardial estimations for different conditions are discussed in Fig. 4. Here, we discuss the MCG forward reconstructions from the estimated heart activities (model 2) and the results are mapped on to the detector planes for field visualization. It was found that there were only small deviations in magnitudes of reconstructions from deterministic and Bayesian methods in noise-free conditions as shown in Fig. 8b–d.

Figure 8f illustrates the reconstruction results of Tikhonov regularization (8 dB SNR) which showed reduction in amplitude range [− 84 27] pT. The MCG waves reconstructed from Bayesian model handled uncertainties and amplitudes appeared near to the range of true maps as shown in Fig. 8g. However, the VBLR reconstructions (Fig. 8h) showed an improvement in the map than Bayesian method and the distribution range found was [− 51 22] pT.

The optimal values of hyperparameters are updated in the simplistic Bayesian modeling by maximizing the measurement likelihood and ratio corresponding to the regularization parameter. The final RMSE measure of Bayesian method in elevated ST case converged to 0.5463 mV, for hyperparameters $\alpha =$ 6.5676 and $\beta =$ 1.907E15 in noise-free condition. For 6dB, the RMSE reached to 2.5081 mV for $\alpha =$ 0.0232 and $\beta =$ 5.38E12. But in VBLR method, the distribution function $q(\alpha )$ known as hyperpriors is approximated using KL divergence. The hyperposterior mean ($E_\alpha (\alpha )=\frac {\hat{c}}{\hat{d}}$) obtained was 0.0023 and ($E_{\beta ,s}(\beta s^\top s)=$ 780.3) at 6 dB (lowest SNR) in ST elevated case. To evaluate the hypers, the optimal ratios of $\frac {\alpha }{\beta }$ are captured at different SNRs along with the converged RMSEs in the hierarchical Bayesian method as tabulated in Table 2. Similarly the means of hyperposteriors are obtained in VBLR technique to study the behaviour at uncertainty conditions. It was observed that there was slight improvement in the RMSE of VBLR method than the Bayesian approach. The nature of variations of $\frac {\alpha }{\beta }$ (hierarchical Bayesian) and $E_\alpha (\alpha )$ (VBLR) with respect to iterations showed similar activities; where the values of both ratio and hyperparameters increase in noisy signals, whereas decrease in noiseless measurements.

**Table 2:** Evaluation of hyper-posterior means with respect to RMSE at different noise levels.

SNR dB	Bayesian		VBLR	
	$\frac {\alpha }{\beta }$	RMSE	$E_{\beta ,s}(\beta s^\top s)$	RMSE
8	4.714E−15	0.6004	1077.9	0.5384
10	2.65E−16	0.547	1083.9	0.5382
12	5.5E−15	0.5463	1084.2	0.5381
14	4.4E−15	0.5463	1084.2	0.5381
16	4.95E−15	0.5463	1084.2	0.5381

## Conclusion

In this paper, we presented inverse problems of MCG using deterministic and probabilistic methods employed on two types of MI cases. The results are estimated in terms of epicardial maps from the observed MCG signals in noisy and noiseless conditions. In competition to the simplistic Bayesian technique, a new method called Variational Bayesian Linear approach is applied which contained non-stationary normal inverse-gamma priors that used KL divergence to approximate the posteriors. The other drawback of selecting the optimal parameter is addressed in Tikhonov regularization. It is shown that the problem is overcome in Bayesian models that learn the posteriors automatically and estimate the unknowns from prior knowledge and observations. The performance of VBLR method is shown to be better than the simplistic Bayesian approach since it uses varying distribution of priors that estimate the posteriors of heart activities which fit well using KL divergence. The current work on inverse algorithms has been tested on different heart shapes. Further, the method assumed zero effect of the conductivity profile to the magnetic measurements in the forward simulation steps, due to which, the transfer matrix was built to map between different heart shapes and the detectors. The complicated electrodynamic design of heart and torso with respiratory movements, skin conductivity profiles, bio-electric and bio-magnetic fields has to be modelled in future studies to mimic a realistic environment for cardiac source localization.

To avoid the inverse crime, two different geometrical models were used. The forward measurements were simulated from the potentials of the first heart model. The observed MCG data was tested on the different heart geometry (model 2) by inverting the forward matrix constructed from the first model in order to determine the source activities.

The current outcome of this research limits its study to the simulations and solving the inverse problems of MCG on different realistic heart models. The future study of this research involves localization of abnormal cardiac sources due to the effect of other artefacts such as realistic noise levels and breathing movements with physically acquired MCG measurements. The results of this paper promise the clinicians an advantage of diagnosing the infarction region or any other cardiac-related diseases as the reconstructed heart surface potential maps on the myocardium. The study provides the advantage of source localization with high accuracy. This serves as an inspiration to design an online system for non-invasive monitoring of inverse solutions during MCG recording where the clinician will be able to view the unknown functional activities on the screen. However, the models depicted in the paper are limited only to generic models. There is a motive to obtain good quality localization for modeling the geometries of sources acquired from the individual Magnetic Resonance Imaging (MRIs) systems. Further, the inverse problems of the cardiac activations presented in this paper from the MCG signals are to be thoroughly investigated for the subject-specific assessments and could be validated with the recorded epicardial potentials of the structurally healthy hearts.

## Data Availability

The datasets generated during the current study are available in the ECGSIM software https://www.ecgsim.org/index.php and the computations were simulated and analysed using SCIRUN https://www.sci.utah.edu/cibc-software/scirun.html.
